# Escalation of liPid-lOwering therapy in patientS wiTh vascular disease receiving HIGH-intensity statins: the retrospective POST-HIGH study

**DOI:** 10.1038/s41598-021-88416-z

**Published:** 2021-04-26

**Authors:** Jaehyung Ha, Bom Lee, Jung Mi Park, Moonjong Kang, Jaewon Oh, Chan Joo Lee, Sungha Park, Seok-Min Kang, Sang-Hak Lee

**Affiliations:** 1 Division of Cardiology, Department of Internal Medicine, Severance Hospital, Yonsei University College of Medicine, 134 Shinchon-dong, Seodaemun-gu, Seoul, 120-752 Korea; 2 Department of Biostatistics and Computing, Graduate School, Yonsei University, Seoul, Korea

**Keywords:** Outcomes research, Cardiology, Risk factors

## Abstract

In this retrospective study, we investigated whether lipid-lowering therapy (LLT) escalation has clinical benefits in patients with atherosclerotic cardiovascular disease (ASCVD) and low-density lipoprotein cholesterol (LDL-C) levels of 55–99 mg/dL (1.4–2.6 mmol/L), post high-intensity. Out of 6317 Korean patients screened in 2005–2018, 1159 individuals with ASCVD and LDL-C levels of 55–99 mg/dL after statin use equivalent to 40 mg atorvastatin were included. After 1:2 propensity score matching, 492 patients (164 with LLT escalation, 328 controls without LLT escalation) were finally analysed. Primary outcome variables were major adverse cardiovascular and cerebrovascular events (MACCE) and all-cause death. At median follow-up (1.93 years), the escalation group had a lower MACCE rate (1.72 vs. 3.38 events/100 person-years; hazard ratio [HR] 0.34, 95% confidence interval [CI] 0.14–0.83; *p* = 0.018) than the control group. The incidence of all-cause death (0.86 vs. 1.02 events/100 person-years; HR 0.58, 95% CI 0.15–2.19; *p* = 0.42) and each MACCE component did not differ between groups. Kaplan–Meier curves exhibited lower risk of MACCE in the escalation group (HR 0.36, 95% CI 0.12–0.97; *p* = 0.040) but a difference not statistically significant in all-cause death (HR 0.30, 95% CI 0.04–2.48; *p* = 0.26). LLT escalation was associated with reduced cardiovascular risk, supporting more aggressive LLT in this population.

## Introduction

The principles of contemporary lipid-lowering therapy (LLT) are based on a volume of evidence showing that stronger LLT can yield greater absolute benefits, particularly in patients with high cardiovascular risk^1^. When a patient carries a very high risk of conditions such as atherosclerotic cardiovascular disease (ASCVD), the majority of the latest guidelines recommend a reduction in the low-density lipoprotein cholesterol (LDL-C) levels to < 70 mg/dL (1.8 mmol/L)^2^ or < 55 mg/dL (1.4 mmol/L)^3^ with a 50% simultaneous reduction in the levels. However, evidence showing the benefits of a further reduction in lipid concentration in patients with LDL-C levels of 55 to 99 mg/dL (1.4–2.6 mmol/L), particularly in those with levels < 70 mg/dL, is scarce.

Despite the evidence and recommendations mentioned above, high-intensity statins and dose escalation are used less often than needed in real-world practice, as shown in many reports worldwide^4–6^. Moreover, suboptimal lipid lowering by insufficient drug doses may elevate the future cardiovascular risk^7^.

It has been reported that the reduction in cholesterol levels despite the use of the same statin doses is sometimes greater in East Asians than in other ethnicities^8^. In addition, a low-intensity statin regimen showed outcome benefits in Japanese individuals^9^, whereas a similar regimen in a study with participants consisting mostly of Caucasians or those of African descendants did not^10^. In this regard, East Asians are considered more sensitive to lipid-lowering drugs. Therefore, physicians managing such populations are hesitant to treat them with high-intensity regimens. Particularly, physicians may be less open to escalating the LLT intensity when the post-treatment LDL-C levels are 55 to 99 mg/dL (1.4–2.6 mmol/L), slightly above the current target levels. However, the availability of research data supporting decision-making on pharmacotherapy in such clinical settings is highly limited.

The aim of this POST-HIGH (Escalation of lipid-lOwering therapy in patientS wiTh vascular disease and HIGH-intensity statins) study was to investigate whether escalation of LLT intensity has clinical benefits in 1159 Koreans with ASCVD and LDL-C levels of 55–99 mg/dL (1.4–2.6 mmol/L) despite the use of statins with an efficacy similar to atorvastatin 40 mg. We used propensity score matching to select patients for the LLT escalation and control groups and compared clinical outcomes between the groups.

## Methods

### Study population

The Institutional Review Board of Severance Hospital, Seoul, Korea, approved this study (No. 4-2020-476). The study protocol conformed to the ethical guidelines of the 1975 Declaration of Helsinki. The subjects were selected from patients who visited the Division of Cardiology, Severance Hospital, Seoul, Korea, between 2005 and 2018. The need for informed consent from the subjects themselves was waived for the following reasons: (1) the research involved no more than minimal risk to the subjects; (2) the waiver did not adversely affect the rights and welfare of the subjects. The waiver was approved by the Institutional Review Board mentioned above.

Consecutive patients who visited the outpatient clinic during the study period were initially screened. The inclusion criteria were as follows: a diagnosis of ASCVD, use of high-intensity statins equivalent to atorvastatin 40 mg, LDL-C levels of 55–99 mg/dL (1.4–2.6 mmol/L) assessed at least 2 months post-treatment with the aforementioned regimen, and a regular follow-up of ≥ 12 months with laboratory work-up. ASCVD included coronary artery disease, ischaemic stroke, peripheral artery disease, carotid artery disease, and aortic disease. Patients meeting all the inclusion criteria were eligible for the study. We excluded patients who used LLT inconsistently or with inconsistent intensity throughout the follow-up period. We defined inconsistent LLT use as prescription of LLT agents for a period < 80% of follow-up duration. Inconsistent LLT intensity was defined as a change in the LLT agent to different intensity categories during the follow-up and the treatment duration with the mainly-prescribed LLT agent comprising < 80% of total prescription period.

To reduce the impact of selection bias and potential confounders, we conducted a 1:2 propensity score matching of patients with an escalated intensity of LLT (escalation group) versus those with a maintained intensity of LLT (control group). Details on the method for matching is described in the statistical analysis section.

### Study protocol

This was a retrospective, propensity score-matched study. Clinical data including demographic variables and medical history were obtained by trained interviewers. Blood samples were collected after a 12-h fast and analysed by the local laboratory, which is certified by the Korean Society of Laboratory Medicine. The data derived from these samples were used retrospectively for this study. All patients received standard medical therapies at the physicians’ discretion. LLT intensity and titration were decided based on domestic-^8^ or major international guidelines^11–13^ frequently followed during the study period. For example, most of the guidelines that physicians referred recommended < 70 mg/dL (1.8 mmol/L) as an LDL-C target for individuals with ASCVD. The statin intensity was defined according to the 2013 American College of Cardiology/American Heart Association guidelines^13^.

Patients were followed-up in the outpatient clinic every 3–6 months; they underwent lipid profile examination every 3–12 months. The baseline LDL-C level was defined as the level under treatment with high-intensity statins before LLT escalation. The follow-up LDL-C level was determined as the average level examined during the follow-up period. The clinical outcome data were obtained by reviewing the medical records and data from the Korean Statistical Information Service. The primary outcome variables were as follows: the rates of major adverse cardiovascular and cerebrovascular events (MACCE) and all-cause death. MACCE was defined as the composite of cardiovascular death, nonfatal myocardial infarction, percutaneous coronary intervention, coronary artery bypass grafting, and nonfatal ischaemic stroke. Cardiovascular death was defined as death resulting from cardiovascular causes, including acute myocardial infarction, sudden cardiac death, heart failure, or stroke^14^. Myocardial infarction was defined according to the following conditions: clinical symptoms, ECG changes, or abnormal imaging findings suggestive of myocardial infarction, along with an increase of creatine kinase myocardial band fraction above the upper limit of normal or an increase of troponin-T/troponin-I above the 99^th^ percentile of the upper limit of normal^15^. Percutaneous coronary intervention or coronary artery bypass grafting were chosen based on (1) diameter stenosis > 50% according to quantitative coronary angiographic analysis with positive stress test results, or (2) diameter stenosis > 70%. Ischemic stroke was detected by new neurological deficit and confirmed using a neurological examination and imaging work-up. When outcome data were collected, the occurrence of events were confirmed according to these definitions. The secondary outcome variables were the individual components of MACCE.

### Statistical analysis

Continuous variables were tested for normality using the Shapiro–Wilk normality test. Variables with normal distribution are presented as means ± standard deviation, and non-normally distributed variables are presented as median (interquartile range: IQR). Categorical data are presented as frequencies and percentages. Clinical and laboratory data were compared using the chi-square test.

Propensity scores were estimated with a multivariable logistic regression including age, sex, hypertension, diabetes mellitus, chronic kidney disease (estimated glomerular filtration rate < 60 mL/min/1.73 m^2^), atrial fibrillation, acute coronary syndrome, body mass index, number of diseased coronary arteries, and LDL-C levels. Following the estimation of score, patients with or without LLT escalation were matched using 1:2 nearest neighbour matching with a caliper distance set at 0.2 standard deviation of the logit of score. The matching and LLT escalation effect estimation were conducted with the “Matching” package for R. Post-matching validation was performed according to the standard mean difference of all baseline covariates using a threshold of 0.1 to indicate imbalance.

After propensity score matching, McNemar’s test for categorical variables was performed to evaluate associations. Continuous variables with non-normal distribution were compared using Wilcoxon signed-rank test, whereas paired t-test was applied to compare normally distributed variables. Conditional logistic regression was utilized to identify the effect of LLT escalation in consideration of propensity-matched pairs. The cumulative survival curves for each group were plotted using the Kaplan–Meier method and compared using stratified Cox’s proportional hazard regression model. All analyses used two-tailed tests with a significance level of 0.05. The statistical software R version 3.3.2 (R Foundation for Statistical Computing, Vienna, Austria) was used for the analyses.

## Results

### Clinical characteristics

**Figure 1:**
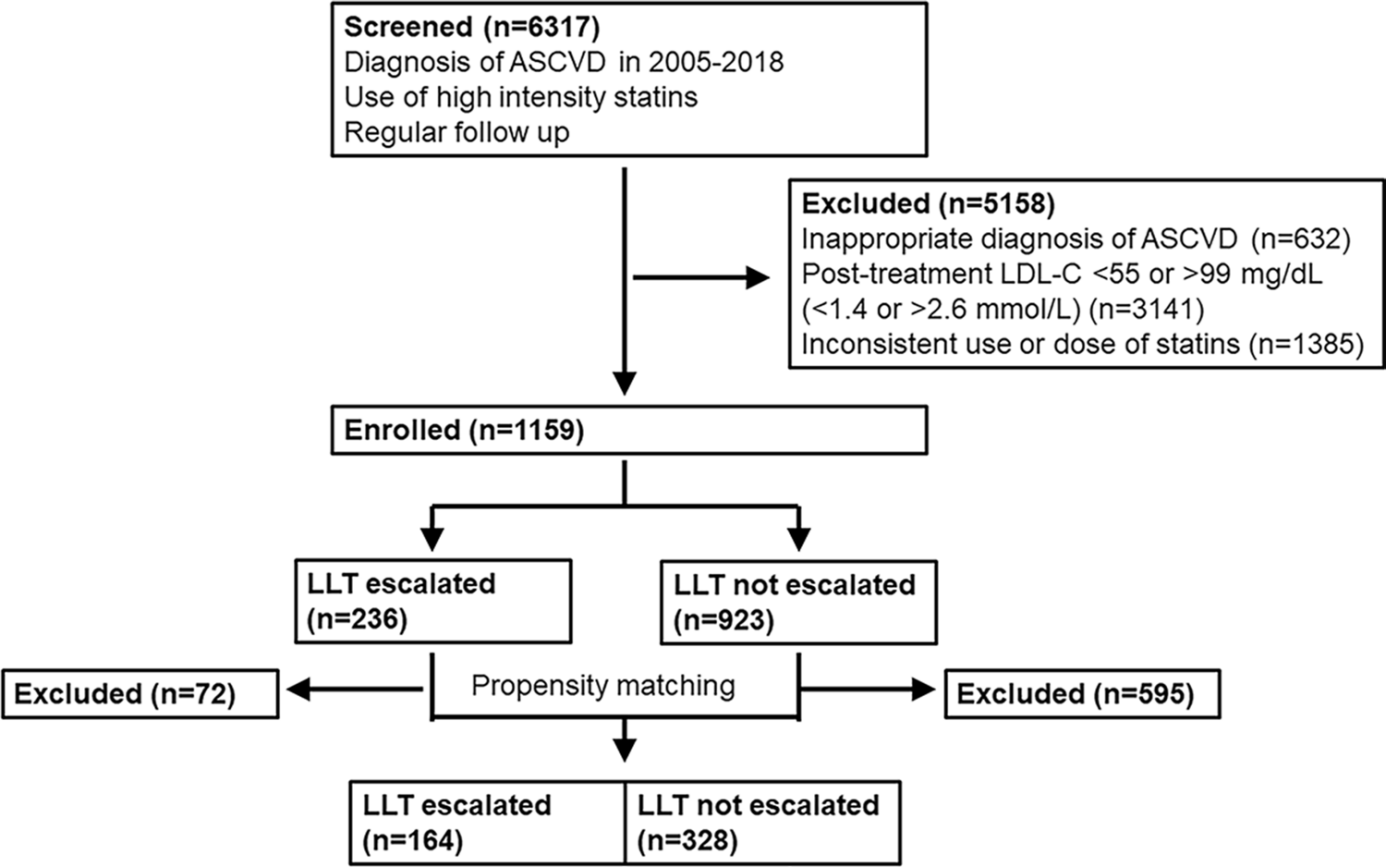
Flowchart of patient inclusion. ASCVD: atherosclerotic cardiovascular disease; LDL-C: low-density lipoprotein cholesterol; LLT: lipid-lowering therapy.

Of the 6317 Korean patients screened, 1159 individuals who met the inclusion criteria with LDL-C levels of 55 to 99 mg/dL (1.4 to 2.6 mmol/L) after statin therapy equivalent to atorvastatin 40 mg/day were enrolled. Among them, 236 patients were prescribed escalated LLT (escalation group) and 923 patients were not (control group). After propensity score matching, 492 patients (164 and 328 in the escalation and control groups, respectively) were finally analysed (Fig. 1). The mean age of the total study sample was 59 years, and 377 patients (77%) were men. Most (92%) individuals were diagnosed with coronary artery disease and the median LDL-C level at enrolment were 80 and 79 mg/dL (2.04 and 2.02 mmol/L) in the escalation and control groups, respectively. The ranges of levels were 55–99 mg/dL (1.42 .56 mmol/L) (Table 1).

**Table 1:** Characteristics of the matched study population.

	Escalation (n = 164)	Control (n = 328)	*p*
Age, years	59.5 ± 10.0	58.8 ± 10.6	0.50
Men	123 (75.0)	254 (7.4)	0.62
Risk factor Hypertension Diabetes mellitus Chronic kidney disease Atrial fibrillation	99 (60.4) 32 (19.5) 8 (4.9) 4 (2.4)	196 (59.8) 68 (20.7) 14 (4.3) 10 (3.0)	0.93 0.77 0.86 0.80
Vascular disease Coronary artery disease Ischaemic stroke Peripheral artery disease Carotid artery disease Aortic disease	146 (89.2) 14 (8.4) 1 (0.6) 2 (1.2) 1 (0.6)	296 (90.4) 27 (8.1) 1 (0.3) 3 (0.9) 1 (0.3)	0.93
Acute coronary syndrome	78 (47.6)	156 (47.6)	> 0.99
Body mass index, kg/m^2^	25.0 (23.4, 26.9)	24.7 (23.2, 26.9)	0.62
Number of diseased coronary vessels 0 1 2 3	7 (4.3) 69 (42.1) 45 (27.4) 43 (26.2)	12 (3.7) 140 (42.7) 91 (27.7) 85 (25.9)	0.98
LDL-C			
Baseline, mg/dL Range, mg/dL	80 (74, 89) (2.07 [1.91, 2.30] mmol/L) 55–98 (1.42–2.53 mmol/L)	79 (73, 88) (2.04 [1.89, 2.28] mmol/L) 55–99 (1.42–2.56 mmol/L)	0.50
Follow-up, mg/dL Range, mg/dL	65 (54, 73) (1.68 [1.40, 1.89] mmol/L) 35–115 (0.91–2.97 mmol/L)	74 (65, 83) (1.91 [1.68, 2.15] mmol/L) 31–144 (0.80–3.72 mmol/L)	< 0.001
Percent change Achievement of < 55 mg/dL (1.4 mmol/L)	-20.9 (-34.3, -6.7) 46 (28.0)	-6.3 (-16.7, 6.8) 20 (6.1)	< 0.001 < 0.001

Each value is presented as mean ± standard deviation or number (%); LDL-C, low-density lipoprotein cholesterol.

In the escalation group, 55% of patients received atorvastatin 40 mg/ezetimibe 10 mg or a similar lipid-lowering regimen and 39% of patients were treated with atorvastatin 20 mg/ezetimibe 10 mg or its equivalent. Detailed information on the equivalent regimens is described in the footnote of Table 2. The majority (76%) of the control group was prescribed rosuvastatin 20 mg and 21% of the same group received atorvastatin 40 mg (Table 2).

**Table 2:** Lipid-lowering regimen used in each group.

	Escalation (n = 164)	Control (n = 328)
Atorvastatin 40 mg	–	68 (20.7)
Rosuvastatin 20 mg	–	249 (75.9)
Simvastatin 10 mg/ezetimibe 10 mg	–	11 (3.4)
Atorvastatin 80 mg or regimen with similar efficacy^a^	8 (4.9)	–
Atorvastatin 20 mg/ezetimibe 10 mg or regimen with similar efficacy^b^	64 (39.0)	–
Atorvastatin 40 mg/ezetimibe 10 mg or regimen with similar efficacy^c^	92 (55.1)	–

Each value is presented as number (%);

^a^: atorvastatin 10 mg/ezetimibe 10 mg, rosuvastatin 5 mg/ezetimibe 10 mg, simvastatin 20 mg/ezetimibe 10 mg, pitavastatin 2 mg/ezetimibe 10 mg;

^b^: rosuvastatin 10 mg/ezetimibe 10 mg, simvastatin 40 mg/ezetimibe 10 mg, pitavastatin 4 mg/ezetimibe 10 mg.

^c^: rosuvastatin 20 mg/ezetimibe 10 mg.

### Clinical outcomes

**Figure 2:**
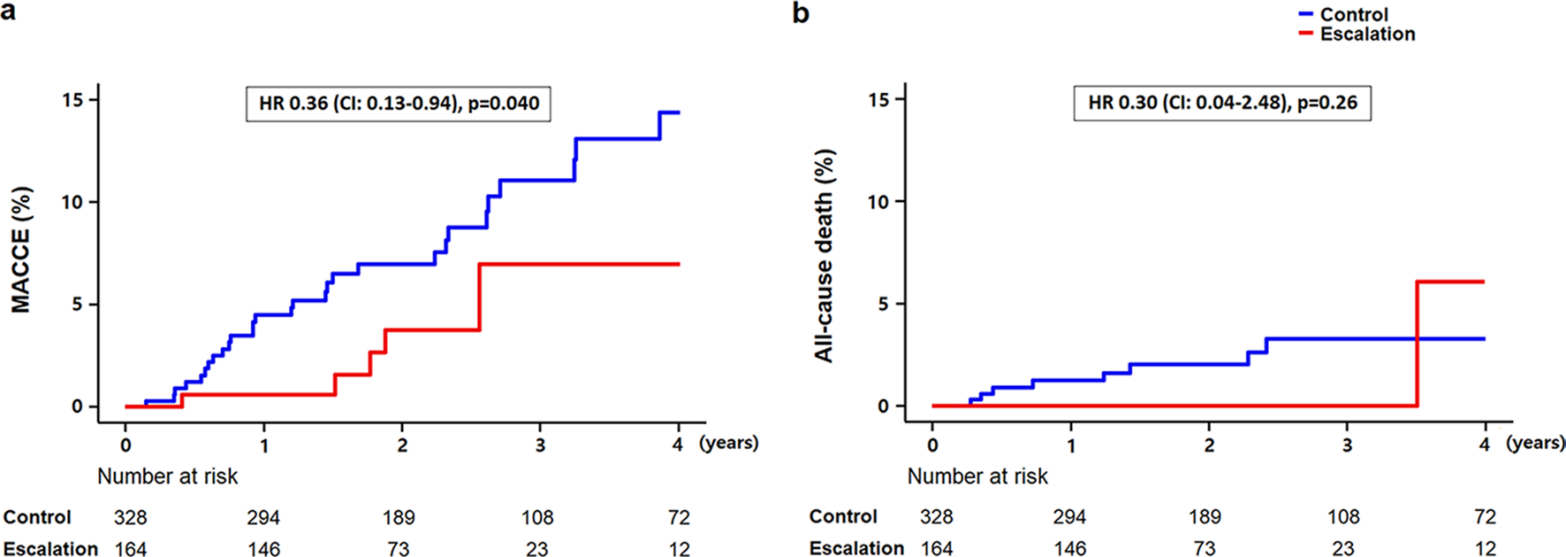
Incidence rates of MACCE (**a**) and all-cause death (**b**). MACCE: major adverse cardiovascular and cerebrovascular events.

The patients were followed-up for a median period of 1.93 years. The median follow-up LDL-C levels were 65 and 74 mg/dL (1.68 and 1.91 mmol/L) in the escalation and control groups, respectively (*p* < 0.001). The ranges of levels were 35–111 mg/dL (0.91–2.87 mmol/L) and 31–144 mg/dL (0.80–4.03 mmol/L) in each group, respectively. The percentage change in the levels was higher in the escalation group (− 20.9 [− 34.3, − 6.7]% and − 6.3 [− 16.7, 6.8]%, respectively, *p* < 0.001) (Table 1). During the follow-up, the escalation group had significantly lower rates of MACCE (1.72 vs 3.38 events/100 person-years; hazard ratio [HR] 0.34, 95% confidence interval [CI] 0.14–0.83; *p* = 0.018) than the control group. The incidence of all-cause death between the two groups did not differ (0.86 vs 1.02 events/100 person-years; HR 0.58, 95% CI 0.15–2.19; *p* = 0.42) (Table 3). The Kaplan–Meier curves exhibited a lower risk of MACCE in the escalation group (HR 0.36, 95% CI 0.13–0.94; *p* = 0.040), whereas the difference in all-cause death was not significant (HR 0.30, 95% CI 0.04–2.48; *p* = 0.26) (Fig. 2). Although the rate of percutaneous coronary intervention was numerically lower in the escalation group, the difference was not significant. Other components of MACCE did not differ between the two groups (Table 3).

**Table 3:** Incidence of events in each group.

	Number of events (events/100 person-year)		HR (95% CI)	*p*
	Escalation (n = 164)	Control (n = 328)		
MACCE	6 (1.72)	33 (3.38)	0.34 (0.14, 0.83)	0.018
Cardiovascular death	0 (0)	1 (0.10)	–	–
Myocardial infarction	0 (0)	4 (0.41)	–	–
PCI	5 (1.43)	24 (2.46)	0.41 (0.15, 1.08)	0.070
CABG	1 (0.29)	0 (0)	–	–
Ischaemic stroke	0 (0)	4 (0.41)	–	–
All-cause death	3 (0.86)	10 (1.02)	0.58 (0.15, 2.19)	0.42

HR, hazard ratio; CI, confidence interval; MACCE, major adverse cardiovascular and cerebrovascular events; PCI, percutaneous coronary intervention; CABG, coronary artery bypass graft.

## Discussion

The major findings of this study are as follows: (1) In Korean patients with ASCVD already on high-intensity statins but with LDL-C levels of 55 to 99 mg/dL (1.4–2.6 mmol/L), escalation of LLT was associated with remarkably reduced cardiovascular risk; (2) The difference in the risk of all-cause death was not statistically different between the two groups. To our knowledge, this study is the first to demonstrate the clear benefits of LLT escalation above high-intensity statins compared to maintaining LLT intensity. Notably, our study may help overcome the paucity of data regarding this issue. Furthermore, our findings may reassure physicians who are reluctant to uptitrate the LLT in patients, especially in East Asians who are already on high-intensity statins.

Our study is the first to show the clinical benefits of very high-intensity LLT mostly with the statin/ezetimibe combination. Except for a few cases who escalate LLT to atorvastatin 80 mg, > 95% of the escalation group took LLT of statin/ezetimibe combination. In particular, the results were derived from a study on East Asian patients in whom this level of LLT has not been well evaluated. Furthermore, the control group in our study used high-intensity statins, which distinguishes it from that in the IMPROVE-IT study^16^. Therefore, our study results provide evidence that may help overcome the paucity of data on the superior effect of high-intensity LLT with the statin/ezetimibe combination compared to high-intensity statin monotherapy. The only studies that have compared the effects of LLT escalation versus controls on high-intensity statins are the FOURIER^17^ and ODYSSEY OUTCOMES trials^18^ with respect to proprotein convertase subtilisin/kexin type 9 (PCSK9) inhibitors. It is worth mentioning that our study analysed the effects of LLT escalation mostly by adding ezetimibe to high-intensity statins. Although the benefit of ezetimibe has remained uncertain for several years^19^, recent clinical trials have shown that adding ezetimibe to statin therapy elicited a reduction in cardiovascular events and greater coronary plaque regression^16,20,21^. In this regard, the latest European guidelines raised the recommendation level of ezetimibe in patients who do not achieve LDL-C goal with maximal tolerable dose statins^3^.

In the FOURIER^17^ or ODYSSEY OUTCOMES trials^18^, the effects of PCSK9 inhibitor-based LLT escalation were not statistically different between subgroups of high-intensity statins and those of lower-intensity statins. However, in the ODYSSEY OUTCOMES trial^18^, the risk reduction by LLT escalation was numerically lower in those using high-intensity statins. In this regard, we need to further investigate whether the long-term effects of LLT escalation are different from the effects of baseline LLT intensity.

The median baseline LDL-C level in our study population was 80 mg/dL (2.07 mmol/L), which was lower than that (93 mg/dL [2.40 mmol/L]) in the IMPROVE-IT study^16^ or that (92 mg/dL [2.38 mmol/L]) in the FOURIER^17^ or ODYSSEY OUTCOMES trials^18^. Particularly, a substantial portion of the population in the present study had levels between 55 and 69 mg/dL (1.4 and 1.8 mmol/L). Such individuals were not regarded as mandatory candidates for LLT before the release of the 2019 European guidelines on LLT^3^. We found that the effect of LLT escalation was not different between patients with a baseline LDL-C level of > 70 mg/dL (1.8 mmol/L) and those with an LDL-C level of < 70 mg/dL. This suggests that an LDL-C level of < 70 mg/dL, a target until recently, may not be sufficient in our study population. This result is in accordance with that of the post hoc analysis of the FOURIER trial, which identified that the addition of evolocumab to maximal- or submaximal-dose statins induced incremental clinical benefits even in patients with an LDL-C level of < 70 mg/dL^22^. We had found a similar result in our earlier study that revealed that statins equivalent or stronger than atorvastatin 20 mg were more beneficial than statins of a lower efficacy in Korean patients with stable coronary artery disease and a baseline LDL-C level of < 80 mg/dL (2.07 mmol/L)^23^.

The incidence of all-cause mortality was not different between the two groups in our study. However, the all-cause mortality rate was only 1.02/100 person-years in the control group, and it would be difficult to obtain a differential benefit with such a low rate by escalating the LLT. Furthermore, because our sample size and follow-up duration were not determined to reveal mortality difference between the groups, our data on this issue may not be a crucial evidence for a negative result. Conversely, in a meta-analysis on the effect of LLT on mortality reduction, the effect was greater when the baseline LDL-C level was higher and the effect was uncertain when the level of LDL-C was < 100 mg/dL (2.6 mmol/L)^24^. Thus, it may be difficult to conclude definitively whether LLT escalation to a very high intensity can reduce the mortality risk in patients with higher LDL-C levels than those observed in our study sample.

Moreover, all our study participants were Koreans. There have long been controversies on the differential safety and optimal dose of statins in Asian patients. The 2018 American guidelines described this issue as follows: Japanese people may be sensitive to statin dosing and show a reduced cardiovascular risk with low-intensity pravastatin. Individuals of several Asian ethnicities showed higher rosuvastatin plasma levels than those seen in Caucasians, and the FDA recommends a lower starting dose in Asians^2^. Thus, it is remarkable that we found clinical benefits of escalation of LLT above high-intensity statins in a population of East Asians.

Difference of median follow-up LDL-C between the two groups was small in our study. Difference in LDL-C between a test and control groups tended to be smaller when we compare higher- and lower intensity LLT groups, particularly when baseline LDL-C was low, like in our or the IMPROVE-IT study^16^. In contrast, these differences were greater when baseline LDL-C was higher like the patients enrolled in the TNT study^25^. Additionally, although the underlying mechanism is not clear, the smaller difference in LDL-C between the study groups could cause greater outcome difference in East Asians like in the MEGA study^9^. Therefore, the smaller difference in LDL-C in our study population with low baseline LDL-C possibly has significant clinical meaning. The hazard ratio of 0.36 for MACCE is more dramatic than estimation by major meta-analysis data^1^. In addition, it is difficult to rule out that biases-related to the observation study design could have affected our results. Conversely, it is not clear whether cardiovascular risk reduction by equivalent LLT is greater in East Asians than in other ethnicities, as trial data are too scarce in East Asians including Koreans. As mentioned above, the MEGA study conducted in Japan reported a hazard ratio for cardiovascular events of 0.67, and this corresponded to a much larger risk reduction than estimated by 15% additional reduction of LDL-C^9^. Furthermore, similar results were found in the EWTOPIA 75 study performed using ezetimibe in Japan^26^. However, it is not clear what underlies the greater benefit in our study than estimated.

Nevertheless, our study has some limitations. First, the mean follow-up duration of 1.93 years is relatively short. Although LLT escalation has shown a significant differential effect in this duration, it is not clear from these results whether the benefit may change in a longer-term follow-up. Second, despite the significant benefits with regard to MACCE, data on drug safety were not available in our study. The availability of safety data might have helped in calculating the net benefit of our LLT strategies and strengthened the conclusions. Third, the difference in the composite endpoint was driven mostly by different percutaneous coronary interventions. Other endpoints occurred too infrequently to help draw any conclusions. Percutaneous coronary intervention is regarded as a softer event and more dependent on physician’s discretion than other end points. Nonetheless, we provide evidence that will be helpful in these specific conditions when clinical decision-making is commonly difficult, and this may be the greatest strength of our study. Fourth, we cannot completely rule out that physicians might have considered coronary intervention less in patients under more aggressive LLT. Furthermore, we could not correct for the prescriber factor during propensity score matching due to insufficient information. Different medical care that is related to prescriber factor might not be fully ruled out.

In conclusion, escalation of LLT was associated with reduced cardiovascular risk in patients with ASCVD who are already on high-intensity statins but with LDL-C levels of 55–99 mg/dL. Our results provide evidence on the need for more aggressive LLT uptitration in patients in whom high-intensity statins alone were considered adequate for treatment.

## Supplementary Information

Supplementary Information

## Data Availability

The datasets generated during and/or analyzed during the current study are available from the corresponding author on reasonable request.

## References

[1] Cholesterol Treatment Trialists’ (CTT) Collaboration. *et al*. Efficacy and safety of more intensive lowering of LDL cholesterol: a meta-analysis of data from 170,000 participants in 26 randomised trials. *Lancet.* **376**, 1670–1681. 10.1016/S0140-6736(10)61350-5 (2010). 10.1016/S0140-6736(10)61350-5 PMC2988224 21067804

[2] Grundy SM (2019). 2018 AHA/ACC/AACVPR/AAPA/ABC/ACPM/ADA/AGS/APhA/ ASPC/NLA/PCNA guideline on the management of blood cholesterol: a report of the American College of Cardiology/American Heart Association Task Force on clinical practical guidelines. J. Am. Coll. Cardiol..

[3] Authors/Task Force Members, ESC Committee for Practice Guidelines (CPG) & ESC National Cardiac Societies. 2019 ESC/EAS guidelines for the management of dyslipidaemias: lipid modification to reduce cardiovascular risk, *Atherosclerosis.* **290**, 140–205. 10.1016/j.atherosclerosis.2019.08.014 (2019). 10.1016/j.atherosclerosis.2019.08.014 31591002

[4] Choi SY, Yang BR, Kang HJ, Park KS, Kim HS (2020). Contemporary use of lipid-lowering therapy for secondary prevention in Korean patients with atherosclerotic cardiovascular diseases. Korean J. Int. Med..

[5] Banefelt J, Lindh M, Svensson MK, Eliasson B, Tai MH (2020). Statin dose titration patterns and subsequent major cardiovascular events in very high-risk patients: estimates from Swedish population-based registry data. Eur. Heart. J. Qual. Care Clin. Outcomes..

[6] Lee SH (2019). Dyslipidemia and rate of under-target low-density lipoprotein-cholesterol in patients with coronary artery disease in Korea. J. Lipid Atheroscler..

[7] Akyea RK, Kai J, Qureshi N, Iyen B, Weng SF (2019). Sub-optimal cholesterol response to initiation of statins and future risk of cardiovascular disease. Heart.

[8] Rhee EJ (2019). 2018 guidelines for the management of dyslipidemia in Korea. J. Lipid. Atheroscler..

[9] Nakamura H (2006). Primary prevention of cardiovascular disease with pravastatin in Japan (MEGA study): a prospective randomised controlled trial. Lancet.

[10] ALLHAT Officers and Coordinators for the ALLHAT Collaborative Research Group. The Antihypertensive and Lipid-Lowering Treatment to Prevent Heart Attack Trial. Major outcomes in moderately hypercholesterolemic, hypertensive patients randomized to pravastatin vs usual care: the antihypertensive and lipid-lowering treatment to prevent heart attack trial (ALLHAT-LLT). *JAMA.* **288**, 2998–3007. 10.1001/jama.288.23.2998 (2002). 10.1001/jama.288.23.2998 12479764

[11] Grundy SM (2004). Implications of recent clinical trials for the National Cholesterol Education Program Adult Treatment Panel III guidelines. Circulation.

[12] Catapano AL (2011). ESC/EAS guidelines for the management of dyslipidaemias: The task force for the management of dyslipidaemias of the European Society of Cardiology (ESC) and the European Atherosclerosis Society (EAS). Atherosclerosis.

[13] Stone NJ (2014). 2013 ACC/AHA guideline on the treatment of blood cholesterol to reduce atherosclerotic cardiovascular risk in adults: a report of the American College of Cardiology/American Heart Association Task Force on Practice Guidelines. Circulation.

[14] Garcia-Garcia HM (2018). Standardized end point definitions for coronary intervention trials: the academic research consortium-2 consensus document. Circulation.

[15] Thygesen K (2018). Fourth universal definition of myocardial infarction. Circulation.

[16] Cannon CP (2015). Ezetimibe added to statin therapy after acute coronary syndrome. N. Engl. J. Med..

[17] Sabatine MS (2017). Evolocumab and clinical outcomes in patients with cardiovascular disease. N. Engl. J. Med..

[18] Schwartz GG (2018). Alirocumab and cardiovascular outcomes after acute coronary syndrome. N. Engl. J. Med..

[19] Katsiki N, Theocharidou E, Karagiannis A, Athyros VG, Mikhailidis DP (2013). Ezetimibe therapy for dyslipidemia: an update. Curr. Pharm. Des..

[20] Tsujita K (2015). Impact of dual lipid-lowering strategy with ezetimibe and atorvastatin on coronary plaque regression in patients with percutaneous coronary intervention: the multicenter randomized controlled PRECISE-IVUS trial. J Am Coll Cardiol..

[21] Pradhan A, Bhandari M, Sethi R (2020). Ezetimibe and improving cardiovascular outcomes: current evidence and perspectives. Cardiol. Res. Pract..

[22] Giugliano RP (2017). Clinical efficacy and safety of evolocumab in high-risk patients receiving statin: secondary analysis of patients with low LDL cholesterol levels and in those already receiving a maximal-potency statin in a randomized clinical trial. JAMA Cardiol..

[23] Lee SY (2016). Statin intensity and clinical outcome in patients with stable coronary artery disease and very low LDL-cholesterol. PLoS ONE.

[24] Navarese EP (2018). Association between baseline LDL-C level and total and cardiovascular mortality after LDL-C lowering: a systemic review and meta-analysis. JAMA.

[25] LaRosa JC (2005). Intensive lipid lowering with atorvastatin in patients with stable coronary disease. N. Engl. J. Med..

[26] Ouchi Y (2019). Ezetimibe lipid-lowering trial on prevention of atherosclerotic cardiovascular disease in 75 or older (EWTOPIA 75): a randomized, controlled trial. Circulation.

