# Supplementary material for: Escalation of liPid-lOwering therapy in patientS wiTh vascular disease receiving HIGH-intensity statins: the retrospective POST-HIGH study
Source: Sci Rep. 2021 Apr 26;11:8884. doi: 10.1038/s41598-021-88416-z (PMC8076278; doi:10.1038/s41598-021-88416-z)
Supplement: Supplementary file 1 — Supplementary Information [file 41598_2021_88416_MOESM1_ESM.pdf]

Escalation of liPid-lOwering therapy in patientS wiTh vascular disease receiving HIGH-intensity statins: The retrospective POST-HIGH study

Jaehyung Ha, Bom Lee, Jung Mi Park, Moonjong Kang, Jaewon Oh, Chan Joo Lee, Sungha Park, Seok-Min Kang, Sang-Hak Lee

Supplementary Methods

R code

```
##Data loading
ex1<-read.csv(file="C:/Users/pookr/Desktop/POST_HIGH.csv")

## Remove of missing values
dt_nomiss<-
ex1 %>%dplyr::select(ID,Dose,Age,Sex,BMI,HTN,DM,CKD,AF,Disease,ACS,CAD,Baseline_LDL,) %>% na.omit()

##Propensity score estimation
dt_psModel<- <-
glm(Dose~Age+as.factor(Sex)+BMI+as.factor(HTN)+as.factor(DM)+as.factor(CKD)+as.factor(AF)+as.factor(Disease)+as.factor(ACS)+as.factor(CAD)+Baseline_LDL+,
family=”binomial”, data=ex1)

##Propensity score matching
library(Matching)
dt_nomiss$pTR <-predict(dt_psModel,type="response")
dt_nomiss$pCN <- 1-dt_nomiss$pTR
```

```
dt_nomiss$pAssign<-NA
dt_nomiss$pAssign[dt_nomiss$Dose == "1"] <- dt_nomiss$pTR[dt_nomiss$Dose == "1"]
dt_nomiss$pAssign[dt_nomiss$Dose == "0"] <- dt_nomiss$pCN[dt_nomiss$Dose == "0"]

dt_listMatch<-Match(Tr=(dt_nomiss$Dose=="1"),
                    X= dt_psModel$fitted.values,
                    M=2,
                    caliper = 0.2,
                    replace=FALSE,
                    ties = TRUE)

dt_TR<- dt_nomiss[unique(dt_listMatch$index.treated),]
dt_CN<- dt_nomiss[dt_listMatch$index.control,]

dt_TR$matched_id <- 1:nrow(dt_TR)
dt_CN$matched_id <- rep(1:nrow(dt_TR),each=2)
dt_total <- rbind(dt_TR, dt_CN)

# Conditional logistic regression for HR
dt_total2 <- left_join(dt_total, ex1, by=’ID’)
con_macce <- clogit(as.numeric(MAACE_01)~Dose.x+strata(matched_id), data= dt_total2, method="exact")
con_death<-clogit(as.numeric(Death)~Dose.x+strata(matched_id),data=dt_total2,method="exact")
con_pci<-clogit(as.numeric(pci)~Dose.x+strata(matched_id), data= dt_total2,method="exact")

# Stratified cox proportional hazard model
cox_macce <- coxph(Surv(Duration, MAACE_01=="1")~Dose.x+strata(matched_id), data= dt_total2)
cox_death <- coxph(Surv(Duration, Death=="1")~Dose.x+strata(matched_id), data= dt_total2)
```
